# Evaluating Free PPV23 Vaccination for the Elderly in Nanning, China: A Cost-Effectiveness Analysis

**DOI:** 10.3390/vaccines13070763

**Published:** 2025-07-18

**Authors:** Zhengqin Su, Linlin Deng, Dan Luo, Jianying Ren, Xiaozhen Shen, Wenjie Liang, Haibin Wei, Xiong Zou, Zhongyou Li, Hai Li

**Affiliations:** 1 School of Public Health and Management, Guangxi University of Chinese Medicine, Nanning 530200, China; suzhengqin2023@stu.gxtcmu.edu.cn (Z.S.); renjianying2024@stu.gxtcmu.edu.cn (J.R.);; 2 Guangxi Key Laboratory of Translational Medicine for Treating High-Incidence Infectious Diseases with Integrative Medicine, Nanning 530200, China; 3 Department of Anesthesiology, First Affiliated Hospital of Guangxi University of Chinese Medicine, Nanning 530023, China; 4 School of Public Health, Guangxi Medical University, Nanning 530021, China; 5 Maternal and Child Health Hospital of Guangxi Zhuang Autonomous Region, Nanning 530002, China

**Keywords:** pneumococcal vaccine, cost-effectiveness analysis, elderly, free vaccination, public health

## Abstract

**Background**: This study aims to evaluate the cost-effectiveness of providing the 23-valent pneumococcal polysaccharide vaccine (PPV23) free of charge versus self-paying vaccination among adults aged 60 years and older in Nanning, Guangxi, China. **Methods**: A decision tree–Markov model was developed to compare three strategies (government-funded free vaccination, self-funded vaccination, and no vaccination) over a 5-year time horizon. The model incorporated local epidemiological data and cost parameters, applying a 3% discount rate. Sensitivity analyses were conducted on key parameters, including vaccine effectiveness against pneumonia and pneumonia treatment costs. **Results**: The benefit–cost ratios for free and self-funded vaccination were 0.075 and 0.015, respectively, both below the cost-effectiveness threshold of 1. However, the free vaccination strategy resulted in a higher net benefit (USD 399,651.32) compared to the self-funded strategy (USD 222,594.14), along with a lower Incremental Cost-Effectiveness Ratio (*ICER*) (USD 1.47 per USD 0.14 of avoided disease cost). Although both strategies yielded benefit–cost ratios far below the conventional threshold of 1, the free strategy demonstrated relatively greater economic efficiency. Sensitivity analyses confirmed that vaccine effectiveness against pneumonia and treatment costs were key drivers of economic outcomes. **Conclusions**: While neither vaccination strategy achieved conventional cost-effectiveness benchmarks in this setting, the free PPV23 vaccination program demonstrated relatively greater economic efficiency compared to the self-funded approach; although neither strategy met the conventional cost-effectiveness thresholds, they should be considered for inclusion in regional health policy for older adults.

## 1. Introduction

*Streptococcus pneumoniae* (Spn) is a common colonizer of the human nasopharynx and is typically non-pathogenic. However, in immunocompromised populations—particularly infants and the elderly—it can breach mucosal defenses, leading to severe invasive diseases such as pneumonia, meningitis, and bacteremia [1,2]. The risk of pneumococcal infection and pneumonia-related mortality rises markedly with age [3]. According to the World Health Organization (WHO), pneumococcal pneumonia causes an estimated one million deaths annually worldwide, with incidence rates among adults aged ≥65 years ranging from 24 to 85 per 100,000 [4]. In China, the hospitalization rate for pneumococcal disease in this age group is nearly three times that of the general population, reaching 200 per 100,000 [5]. Elderly individuals with chronic comorbidities or impaired immunity face an even higher risk of infection, rapid disease progression, and poorer outcomes, often resulting in complications, long-term sequelae, or death—substantially diminishing quality of life and health status. While pneumococcal colonization and mild infections may be more common in younger individuals, older adults experience substantially higher rates of hospitalization, complications, and mortality due to pneumococcal disease, making them the primary target for vaccination programs.

In 2017, WHO estimated that lower respiratory infections and meningitis accounted for 106 million and 20.4 million disability-adjusted life years (DALYs), respectively, with 10–30% of this burden attributed to Spn infection [6]. Preventing invasive pneumococcal disease (IPD) in older adults remains a global public health priority. The advent of antibiotics and vaccines has significantly improved survival rates among pneumonia patients [7], and WHO ranks pneumococcal disease alongside malaria as a “very high priority” for vaccine-preventable intervention [8].

The 23-valent pneumococcal polysaccharide vaccine (PPV23) provides coverage against 23 of the most common Spn serotypes, accounting for 60–85% of disease-causing strains [9,10]. A WHO survey of 194 member states revealed that 163 have incorporated at least one pneumococcal vaccine into their national immunization schedules; among them, 44 include PPV23, primarily in high-income countries such as the United States and the United Kingdom [11]. Despite approval for clinical use in China since 1996, PPV23 remains a self-paid, non-program vaccine with low uptake due to limited healthcare resources [10].

Nanning, the capital of Guangxi Zhuang Autonomous Region, is a model city for integrated elderly care, with a favorable climate, supportive policy infrastructure, and ongoing development of age-friendly environments [12,13]. According to the Guangxi Statistical Yearbook 2023, as of the end of 2022, Guangxi had 8.81 million residents aged ≥60, of whom 1.37 million (15.53%) resided in Nanning [14]. To reduce the pneumococcal disease burden among older adults and protect population health, local health authorities in Guangxi plan to implement a publicly funded single-dose PPV23 vaccination program for residents aged 60 and above. However, given fiscal constraints, a rigorous cost-effectiveness analysis is needed to inform policy decisions.

This study aims to evaluate the cost-effectiveness of a free PPV23 vaccination program for older adults in Nanning from a societal perspective, using a decision tree–Markov model to simulate long-term health and economic outcomes. The findings are intended to provide robust scientific evidence to support policy formulation and resource allocation for this public health initiative.

## 2. Materials and Methods

### 2.1. Decision Tree–Markov Model

The decision tree–Markov model [15] combines two established frameworks for health economic evaluation: the decision tree, which facilitates the visualization of sequential choices; and the Markov model, which enables the simulation of stochastic state transitions over time. A standard decision tree consists of decision nodes, chance nodes, and terminal outcome nodes. It is widely used to determine optimal strategies by calculating the expected benefits or costs associated with different pathways under uncertainty.

The Markov model [16], characterized by its “memoryless” property, assumes that the probability of transitioning to a future state depends solely on the current state, irrespective of the historical trajectory. This structure is particularly well-suited for modeling chronic conditions and long-term interventions where disease progression or recovery follows a probabilistic pattern. The model simulated annual transitions over a 5-year time horizon, reflecting the expected duration of vaccine-induced protection and aligning with typical public health planning cycles.

The integration of these two models—referred to as a decision tree–Markov model—has become a cornerstone in medical decision analysis and health technology assessment. It is especially valuable for evaluating long-term impacts of vaccination strategies and optimizing healthcare resource allocation [15,16]. The approach combines the intuitive logic and visualization of decision trees with the dynamic simulation capabilities of Markov processes, enabling comprehensive analysis of complex health interventions.

### 2.2. Target Population

The target population for this study comprises residents aged 60 years and older in Nanning, Guangxi, as of 2022. All analyses were conducted from a societal perspective to capture the broad economic and health implications of the intervention.

### 2.3. Model Structure and Assumptions

A decision tree–Markov model was constructed using TreeAge Pro Healthcare 2022 R1.2 software to compare the cost-effectiveness of a publicly funded PPV23 vaccination strategy against the current self-paid vaccination approach.

The PPV23 vaccination protocol generally consists of a single dose. A booster dose is recommended only for specific high-risk groups—such as individuals with functional or anatomical asplenia or immunocompromising conditions—with a minimum interval of five years between the primary and booster doses [17]. The model incorporates both the public-funded and out-of-pocket strategies, each with two options: vaccination with a single dose or no vaccination.

**Figure 1:**
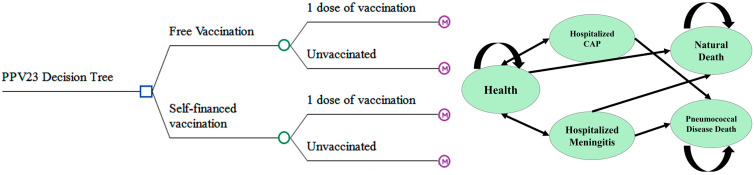
Decision Tree–Markov Models.

The Markov component includes five mutually exclusive health states: healthy, hospitalized community-acquired pneumonia (CAP), hospitalized pneumococcal meningitis, pneumococcal-related death, and all-cause mortality (Figure 1).

The model assumes a 1-year Markov cycle length and simulates health and economic outcomes over a 5-year time horizon, which corresponds to the generally accepted duration of immunological protection provided by a single dose of PPV23. This time frame aligns with existing evidence on vaccine efficacy and local policy planning cycles. Nonetheless, we acknowledge that this relatively short horizon may underestimate potential long-term societal and health benefits beyond the protection period, such as reduced morbidity, caregiver burden, or delayed mortality.

### 2.4. Model Parameters (Key Model Parameters and Data Sources Are Summarized in Table 1)

#### Demographic and Epidemiological Parameters

The model population comprised all residents aged 60 years and older in Nanning by the end of 2022, totaling 1,367,900 individuals; demographic data were sourced from the Guangxi Statistical Yearbook 2023, published by the Guangxi Zhuang Autonomous Region Bureau of Statistics [14].

Whenever possible, model inputs were derived from local data specific to Guangxi. In the absence of local evidence, data from neighboring provinces with comparable geographic, climatic, and dietary characteristics—such as Guangdong, Hunan, Guizhou, and Yunnan—were prioritized to enhance the model’s external validity.

Incidence estimates for community-acquired pneumonia and pneumococcal meningitis were based on population-based surveillance data and the previously published literature on pneumococcal disease burden in Guangxi. The incidence of CAP was derived from three key sources: a prospective multicenter study across 14 hospitals (889 cases), surveillance data from two sentinel hospitals (999 cases), and a cross-sectional study in Baise City (130 cases). These data were combined and weighted to account for regional and demographic variability. For pneumococcal meningitis, incidence estimates were based on four surveillance datasets collected between 2006 and 2014, comprising 102 clinically confirmed cases and 1424 syndrome-based cases. Age-stratified incidence distributions were assumed as follows: 0–14 years (19.0%), 15–59 years (63.2%), and ≥60 years (17.8%). A stratified weighted estimate was calculated using published incidence assumptions for children (0.71 per 100,000) and adults (1.06 per 100,000), resulting in an overall population incidence of 0.94 per 100,000 (95% CI: 0.5–5.0), and an estimated incidence of 0.11–0.52 per 100,000 among older adults [18,19,20,21,22,23,24,25].

Mortality rates were obtained from the China Health Statistical Yearbook 2023 [26].

Multiple data sources were systematically integrated and optimized using statistical modeling techniques to reduce bias and enhance the robustness of the estimates. The serotype coverage rate of PPV23 was informed by immunogenicity and effectiveness data from studies conducted in Guangdong Province [27].

The model accounted for several dynamic factors, including waning immunity five years post-vaccination, serotype replacement among circulating *Streptococcus pneumoniae* strains, and the potential impact of herd immunity on disease incidence [28]. It was assumed that all cases of meningitis and severe pneumococcal pneumonia required hospitalization, and that all fatal cases occurred in hospital settings. Out-of-hospital deaths due to outpatient-treated CAP were not included, consistent with the scope of mortality data in the China Health Statistical Yearbook 2023 [26]. Natural mortality rates were derived from the Guangxi Statistical Yearbook 2023 [14].

**Table 1:** Parameters of the decision tree–Markov model for cost-effectiveness analysis of free vaccination in PPV23.

Parameter	Value	Range	Source
**Demographic and Epidemiological Parameters**			
Population aged ≥60 in Nanning in 2022 (×10^4^)	136.79	-	Guangxi Statistical Yearbook 2023 [14]
Natural mortality rate in Nanning in 2021 (‰)	6.25	-	Guangxi Statistical Yearbook 2023 [14]
Incidence of community-acquired pneumonia (/100,000)	38	25–45	Literature [18,19,20]
Pneumonia mortality rate (/100,000)	11.22	11.22	China Health Statistics Yearbook 2023 [26]
Meningitis incidence rate (/100,000)	0.21	0.11–0.52	Literature [21,22,23,24,25]
Meningitis mortality rate (/100,000)	0.08	-	China Health Statistics Yearbook 2023 [26]
Serotype coverage of PPV23 against pneumococcal disease (%)	96.6	-	Literature [27]
**Cost Parameters (RMB)**			
Hospitalization cost for pneumonia	10,250	8500–12,000	Literature [29,30,31]
Hospitalization cost for meningitis	39,974	21,836–46,825	Literature [32]
Income loss due to family caregiver leave	120	-	Guangxi Statistical Yearbook 2023 [14]
Cost of severe adverse event	7155.21	±25%	Literature [33] 2022 China Health Statistics Yearbook [34]
Incidence of severe adverse events (/100,000)	0.53	-	National Adverse Drug Reaction Monitoring Center [35]
Vaccine price per dose	166	-	Local data
Cost of vaccination consumables per dose	32	-	Guangxi Public Resource Trading Center [36]
Vaccine wastage rate (%)	1	1.3–1.68	Literature [33]
**Vaccine-Related Parameters**			
Single-dose PPV23 efficacy against pneumonia (%)	77.3	62.8–91.8	Literature [27]
Single-dose PPV23 efficacy against meningitis (%)	59	44–70	Literature [37]
Vaccination coverage rate for free PPV23 dose (%)	13.43	13.43–14.13	Literature [38,39]
Vaccination coverage rate for self-paid PPV23 dose (%)	3.85	1.9–5.8	Literature [38,40,41,42]
**Other Parameters**			
Discount rate (%)	3	3–5	Literature [43,44]

### 2.5. Economic Evaluation Metrics

This study employed the Net Benefit (*NB*) and Benefit–Cost Ratio (*BCR*) as primary indicators to evaluate the economic impact of providing the 23-valent pneumococcal polysaccharide vaccine (PPV23) free of charge. The respective formulas are as follows [16]:

$$NB=B-C=\overset{n}{\underset{t=1}{\sum }}\frac{B_{t}-C_{t}}{{\left(1+r\right)}^{t}}$$

$$BCR=\frac{B}{C}=\frac{\sum_{t=1}^{n}\frac{B_{t}}{{\left(1+r\right)}^{t}}}{\sum_{t=1}^{n}\frac{C_{t}}{{\left(1+r\right)}^{t}}}$$

where *B* denotes the total discounted benefits, defined as the cumulative present value of pneumococcal disease (PD)-related costs averted under the free vaccination strategy compared with out-of-pocket vaccination; *C* represents the total discounted costs, including expenditures for vaccine procurement, administration, and programmatic services; *t* is the year of analysis; *n* is the total time horizon of the program (in years); and *r* is the annual discount rate used to convert future economic outcomes into present value.

An intervention is considered economically favorable if *NB* > 0 or *BCR* > 1.

Additionally, the *ICER* was calculated to compare the additional cost required to achieve each additional unit of monetary benefit (i.e., disease cost averted) when switching from out-of-pocket to publicly funded vaccination:

$$ICER=\frac{Cost_{Free}-Cost_{Out\;of\;Pocket}}{Benefit_{Free}-Benefit_{Out\;of\;Pocket}}$$

Although traditionally the term *“ICER”* refers to cost per unit of health outcome, e.g., Quality-Adjusted Life Year (QALY) gained, our analysis adopts a cost–benefit framework, expressing both costs and benefits in monetary units.

Note: In this study, ‘benefit’ refers to the total discounted monetary value of pneumococcal disease-related costs averted (in USD), rather than health outcomes such as QALYs or cases averted. Thus, this *ICER* represents a monetary-based cost–benefit efficiency metric, and differs from the conventional cost-effectiveness ratio used in QALY-based evaluations.

### 2.6. Sensitivity Analysis

To assess the robustness of the model outcomes, one-way sensitivity analyses were conducted on key parameters. Each analysis involved varying a single input parameter within its plausible range while holding all other parameters constant, in order to evaluate the impact of parameter uncertainty on net benefit outcomes.

## 3. Results

A decision tree–Markov model was employed to evaluate the cost-effectiveness of three pneumococcal vaccination strategies for older adults aged 60 years and above: (1) government-funded single-dose PPV23 vaccination, (2) self-paid single-dose PPV23 vaccination, and (3) no vaccination. The model simulated a 5-year time horizon with an annual discount rate of 3%, using a cohort of 1,367,900 elderly individuals. Based on estimated vaccine uptake rates under each strategy, approximately 183,806 individuals were projected to receive the vaccine under the government-funded strategy, compared with 52,684 under the self-paid strategy.

In terms of total program costs, the government-funded vaccination strategy incurred USD 5.33 million, consisting of USD 4.26 million for vaccine procurement, USD 0.82 million for cold chain and consumables, and USD 0.26 million for service delivery. The self-paid strategy incurred a total cost of USD 1.53 million, including USD 1.22 million for vaccine procurement, USD 0.24 million for consumables, and USD 0.07 million for service delivery.

With respect to health outcomes, based on the modeled incidence rates of pneumonia and meningitis, the government-funded strategy was projected to avert approximately 54 cases of pneumonia and 0.2 cases of meningitis annually. The self-paid strategy would avert 15 cases of pneumonia and 0.06 cases of meningitis annually. When combined with disease treatment costs and discounted to present value, the total economic benefit was estimated at USD 0.4 million for the government-funded strategy and USD 0.22 million for the self-paid strategy.

As summarized in Table 2, the net benefit of the government-funded strategy was USD −4.93 million with a *BCR* of 0.075, while the self-paid strategy yielded a net benefit of USD −1.51 million and a *BCR* of 0.015. The *ICER* comparing government-funded to self-paid vaccination was USD 1.47 per USD 0.14 of disease cost averted.

Although neither vaccination strategy achieved a *BCR* > 1, indicating that they were not cost-saving under the current assumptions, the government-funded strategy demonstrated higher total benefits and a more favorable *BCR* than the self-paid approach. The lower *ICER* further suggests that, despite the absence of overall cost-effectiveness, the government-funded strategy represents a more economically efficient use of resources compared to self-paid vaccination in this population context.

### Sensitivity Analysis Results

A one-way sensitivity analysis was conducted to assess the influence of key parameters on the cost-effectiveness of both government-funded and self-paid PPV23 vaccination strategies. The results identified the vaccine efficacy against pneumonia and the cost of pneumonia treatment as the most influential parameters affecting model outcomes.

When the protective efficacy against pneumonia was increased from 62.8% to 91.8%, the *BCR* for the government-funded strategy rose by 45.9% (from 0.061 to 0.089), while that for the self-paid strategy increased by 38.1% (from 0.063 to 0.087). Correspondingly, the net economic loss decreased by USD 0.15 million and USD 35,843.79, respectively. Similarly, when the treatment cost of pneumonia rose from USD 1185.5 to USD 1673.64, both strategies exhibited a *BCR* increase of over 40%, with reductions in net economic loss of USD 120,641.56 and USD 33,333.33, respectively.

The model was moderately sensitive to changes in the discount rate. An increase in the rate from 3% to 5% led to a 14.5–14.8% decrease in *BCR* for both strategies and resulted in an increase in net economic loss of USD 0.2 million for the government-funded strategy and USD 22,873.08 for the self-paid strategy.

In contrast, the parameters related to meningitis—including vaccine efficacy and treatment cost—had negligible effects on the model outputs. The *BCR* fluctuated by no more than 0.002, indicating minimal sensitivity to these variables.

**Figure 2:**
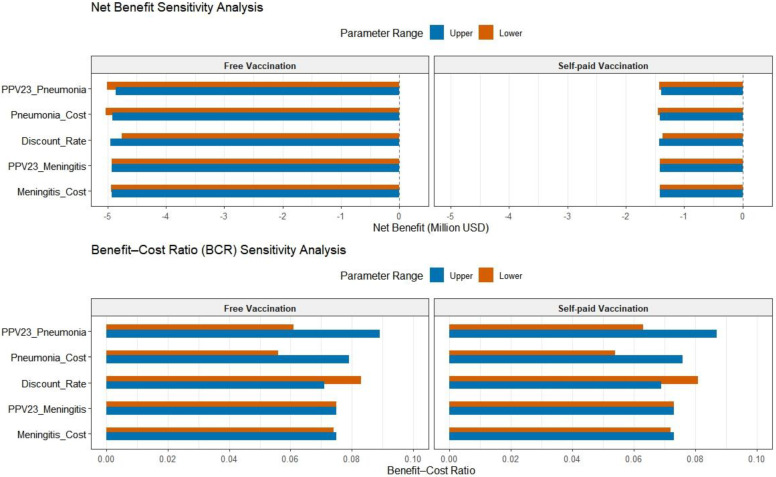
Results of one-way sensitivity analysis for key parameters.

Overall, the sensitivity analysis highlights that, while both pneumonia and meningitis-related parameters were tested, improvements in PPV23’s protective efficacy against pneumonia and better control of pneumonia treatment costs had the greatest impact on economic outcomes. Given the minimal impact of meningitis-related parameters, their role in the model can be simplified. A 4% discount rate is recommended as a baseline for future analyses. In long-term evaluations, priority should be given to calibrating parameters identified as highly sensitive. Detailed results of the sensitivity analysis are presented in Figure 2.

## 4. Discussion

This study evaluated the cost-effectiveness of implementing a government-funded 23-valent pneumococcal polysaccharide vaccine program for adults aged 60 years and older in Nanning, China. Pneumococcal disease—particularly pneumonia—poses a substantial health threat to older populations and has been classified by the World Health Organization as a disease with the highest priority for vaccine-based prevention [8]. While many countries have integrated PPV23 into their national immunization schedules [11], China continues to classify it as a self-paid vaccine, resulting in suboptimal coverage rates [10]. To enhance health outcomes among older adults, the Guangxi Zhuang Autonomous Region initiated a pilot program offering PPV23 free of charge to seniors in Nanning. Given the constraints of limited healthcare resources, this study employed a decision tree–Markov model from a societal perspective to assess the cost-effectiveness of the program and provide evidence-based guidance for policy formulation.

Over a five-year time horizon, the benefit–cost ratios of both the publicly funded and self-paid vaccination strategies were below 1 (0.075 and 0.015, respectively), indicating that neither strategy meets conventional cost-effectiveness thresholds under current parameter assumptions. However, the net present value of benefits was higher for the public program (USD 0.4 million vs. USD 0.22 million), and the *ICER* was lower (USD 1.47 per USD 0.14 of disease cost averted), suggesting that despite being suboptimal in absolute terms, the public vaccination strategy is relatively more cost-effective.

### 4.1. Public Health Impact and Herd Immunity Potential

From the perspective of disease burden, the free vaccination strategy is projected to avert 54 cases of pneumonia and 0.2 cases of meningitis annually, while the self-paid vaccination strategy would prevent only 15 cases of pneumonia and 0.06 cases of meningitis. This disparity is primarily attributable to the significantly higher vaccination coverage. The free vaccination strategy is expected to cover 13.43% of the target population (183,806 individuals), which is substantially greater than the 3.85% coverage (52,684 individuals) under the self-paid strategy.

Studies have demonstrated a strong correlation between vaccination coverage and cost-effectiveness. For instance, Xu et al. [45] used a Susceptible-Exposed-Infectious-Asymptomatic-Recovered-Removed (SEIARR) dynamic model to simulate the impact of increasing influenza vaccination coverage from 50% to 90%. Their findings indicated that the cost-effectiveness ratio dropped from USD 19.67 to USD 15.75, and the benefit–cost ratio increased from 6.27 to 7.88, highlighting that improved vaccination coverage directly optimizes health economic outcomes. Similarly, Ozawa et al. [46] observed that in low- and middle-income countries, each 10% increase in vaccine coverage led to a 15–20% reduction in the cost per disability-adjusted life year averted, with over 52% of vaccination programs achieving a cost of less than USD 100 per DALY, underscoring the economic advantages of high vaccination coverage.

The implementation of free vaccination policies has significantly enhanced vaccination willingness by reducing economic barriers, thereby amplifying herd immunity effects. A study by Kumar et al. [47] utilizing a hybrid strategy model found that combining free vaccination with optimized treatment strategies resulted in an over 40% improvement in economic efficiency compared to single interventions. The indirect protective effects of herd immunity help reduce the infection risk in unvaccinated populations, subsequently lowering overall healthcare expenditures. For instance, an economic evaluation of pneumococcal vaccination conducted by Kim et al. [48] in The Gambia revealed that once vaccination coverage exceeded 70%, hospitalization rates in unvaccinated individuals dropped by 23%, with herd immunity contributing approximately 30% to the total health benefits. While childhood PCV vaccination coverage in developed countries typically exceeds 90%, certain regions in China report coverage rates below 60%. This low coverage may hinder nasopharyngeal colonization blockade and perpetuate the transmission of non-vaccine serotypes [49].

It is noteworthy that meningitis-related parameters exert minimal influence on model outcomes (with *BCR* fluctuations not exceeding 0.002). This may be attributed to the exceptionally low incidence of meningitis in Guangxi (0.11–0.52 per 100,000 in individuals aged ≥60), coupled with the significantly lower protective efficacy of PPV23 against meningitis (59%) compared to pneumonia (77.3%). Therefore, in resource-constrained settings, public health decisions may prioritize the prevention of pneumonia over striving for broad coverage of all PD.

### 4.2. Equity of Free Vaccination

The implementation of a free PPV23 vaccination policy has alleviated, to some extent, the vaccination burden on elderly individuals from economically disadvantaged backgrounds. Many elderly individuals are unable to afford the cost of vaccines, and the free vaccination policy provides them with equitable access to immunization. This is especially important in settings with limited healthcare resources, where the policy plays a critical role. Vaccine-related out-of-pocket costs represent a significant barrier for elderly individuals—59% of this population would refuse vaccination if required to pay for it themselves [50]. By eliminating financial barriers, the free vaccination policy significantly enhances immunization equity. For instance, following the implementation of a free influenza vaccination program in Beijing, vaccination rates showed a significant skew toward the impoverished, with a CI of −0.115, highlighting the largest increases in coverage among low-income and rural elderly populations [51]. This mechanism is of key importance in settings with limited healthcare resources. In Italy, following the introduction of free vaccination for individuals aged 65 and older, vaccination rates surged by 70–90%, and emergency hospitalization rates declined substantially, effectively alleviating the strain on the healthcare system [52].

In resource-constrained areas, the fairness of healthcare services is of paramount importance. The free vaccination policy ensures that all elderly individuals can access vaccine protection, mitigating immunization inequities caused by economic factors. This policy not only improves the health of the elderly population but also helps reduce wealth disparities, thereby enhancing health equity for the entire population.

Our study findings indicate that, while both the free and self-paid PPV23 vaccination strategies have benefit–cost ratios below 1, the free vaccination strategy demonstrated a higher net present value and *BCR*, with a lower *ICER*, suggesting it is more cost-effective. This aligns with the experiences of other economically advanced regions in China, such as Shanghai and Beijing, where pneumococcal vaccination programs have been integrated into free vaccination initiatives [53,54]. These regions have achieved significant increases in elderly vaccination rates, effectively reducing the incidence and mortality of pneumonia and meningitis. However, these areas benefit from stronger economic capacity and more abundant healthcare resources, allowing them to absorb higher vaccination costs, which likely results in more favorable cost-effectiveness outcomes. The Gini coefficient—a statistical measure of inequality, ranging from 0 (perfect equality) to 1 (complete inequality)—for the distribution of high-quality medical resources in China reached 0.6 in 2020, indicating significant regional disparity. The resource density in the eastern regions was three times higher than that in the western regions. Vaccine coverage remains constrained by weak primary healthcare infrastructure and limited fiscal investment [55]. In contrast, the study region has relatively limited economic conditions and healthcare resources. Therefore, while the cost-effectiveness of the free vaccination strategy is lower than in wealthier regions, it remains superior to the self-paid strategy. If successfully implemented, this free vaccination program would contribute significantly to public health prevention for the elderly population.

### 4.3. Policy Discussion Based on the Literature: Cost-Effectiveness of PPV23 Versus PCV13

The 13-valent Pneumococcal Conjugate Vaccine (PCV13) is primarily used for children under the age of 5, preventing diseases caused by 13 serotypes of *Streptococcus pneumoniae* [56]. In contrast, PPV23 is recommended for individuals aged 2 years and older, with a particular focus on those aged 60 and above. Although PCV13 has shown significant effectiveness in preventing pneumonia and meningitis, PPV23 has a broader application in elderly populations. Particularly in middle- and low-income countries such as China, PPV23 offers a greater public health value due to its lower cost [57].

Regarding PCV13, it is recommended for children to receive four doses, while adults, especially the elderly, are advised to receive one dose [58]. The procurement price of the Pfizer PCV13 in the United States is USD 103 per dose (approximately CNY 716), while alternative lower-cost options from manufacturers such as Walvax (Zhifei Biological, Chongqing, China) remain priced at USD 86 per dose (around CNY 598) [59]. This price point limits its accessibility in low-income regions. In comparison, PPV23 provides a more cost-effective form of protection, especially in economically disadvantaged areas. Studies have shown that even with lower vaccination coverage, PPV23 can effectively prevent pneumonia and offers higher cost-effectiveness in resource-limited environments.

International research demonstrates that integrating pneumococcal vaccines into immunization programs yields significant health and cost-effectiveness benefits. Since 2014, the United States has adopted a dual vaccination strategy for individuals aged 65 and older, using PCV13 in conjunction with PPV23 to create a dual immunological barrier. After the introduction of PCV7 (2000–2006), hospitalizations for pneumococcal pneumonia across all age groups decreased by 788,000 annually, with 90% of these benefits attributed to the immunization effects in the adult population. The subsequent use of PCV13 further reduced the incidence of invasive pneumococcal disease in the elderly by 54% [60,61,62,63]. In the United Kingdom, although the cost-effectiveness of vaccination in high-risk populations remains contentious due to serotype replacement (with *ICER*s ranging from GBP 37,216 to GBP 48,210 per QALY), vaccine formulations have been dynamically adjusted to maintain immunization effectiveness [64,65]. Australia’s long-term vaccination program has prevented an estimated 1.77 million cases of disease, with an *ICER* as low as AUD 3347 per QALY, underscoring its public health value [66].

In contrast, the region under study faces significant economic and healthcare resource constraints (e.g., a vaccination coverage rate below 60% in primary healthcare settings and high vaccine price sensitivity), which results in benefit–cost ratios of only 0.075 and 0.015 for the free and self-paid vaccination strategies, respectively, significantly lower than international benchmarks.

This study recommends optimizing existing strategies by drawing from international experience; for example, prioritizing the increase in vaccination coverage in children to trigger herd immunity (with each 10% increase in childhood vaccination coverage reducing adult IPD incidence by 8%); reducing vaccine prices by 30–40% through centralized procurement; and establishing a serotype monitoring network to facilitate vaccine updates (such as introducing PCV15/PCV20 to cover prevalent local serotypes, such as 19A and 3). A long-term system combining “high vaccination coverage among the elderly + broad-spectrum vaccines + tiered fiscal support” should be developed locally, gradually narrowing the cost-effectiveness gap with internationally advanced standards.

### 4.4. Analysis of Key Factors Influencing Cost-Effectiveness Results

The results of the one-way sensitivity analysis in this study indicate that the effectiveness of PPV23 in preventing pneumonia and the treatment costs for pneumonia are the most sensitive parameters affecting the cost-effectiveness outcomes. This is consistent with previous studies. For example, a Markov model-based study showed that the prevention efficacy of PPV23 against invasive pneumococcal disease, particularly serotype coverage, significantly impacts the *ICER*. When vaccine efficacy decreased by 10%, the *ICER* increased from EUR 17,065/QALY to EUR 21,000/QALY, confirming that vaccine efficacy is a key sensitive parameter. Additionally, for every 20% increase in pneumonia treatment costs (e.g., hospitalization expenses), the *ICER* decreased by 15%, highlighting the close relationship between treatment costs and the economic viability of vaccination [67]. A study conducted in the Netherlands also found that the cost-effectiveness of PPV23 for individuals aged 65 and older is highly sensitive to pneumonia incidence and treatment costs. A 30% reduction in pneumonia treatment costs could lead to a 22% decrease in the net benefits of PPV23 [68]. Therefore, enhancing the vaccine’s protective efficacy against pneumonia and implementing dynamic monitoring and control of pneumonia treatment costs are crucial for improving the cost-effectiveness of the PPV23 vaccination strategy.

Furthermore, this study found that the discount rate has a moderate impact on the cost-effectiveness analysis results. It is recommended to use a 4% discount rate as the baseline and prioritize the calibration of highly sensitive parameters in long-term evaluations. This aligns with findings from a Dutch study, which also used a 4% discount rate as the baseline. Sensitivity analysis revealed that when the discount rate fluctuated between 3% and 5%, the *ICER* change was moderate (±12%) [68].

### 4.5. Limitations

This study has several limitations that warrant consideration. First, the regional representativeness of the input data is limited. The incidence of meningitis was extrapolated from data in neighboring provinces such as Guangdong and Hunan, potentially underrepresenting the unique epidemiological dynamics of Guangxi’s border areas, particularly those adjacent to Vietnam, where cross-border transmission of pathogens poses an elevated risk.

Second, the model assumes a simplified linear waning of vaccine-induced immunity over a five-year period. However, empirical evidence suggests that antibody titers may decline in an exponential fashion, particularly under high-temperature environmental conditions. This simplification may result in an overestimation of vaccine effectiveness in real-world settings.

Third, the model does not capture potential long-term health benefits associated with vaccination, such as improvements in quality of life or reductions in non-medical (e.g., caregiving or productivity loss) costs. This omission could lead to an underestimation of the true economic value of vaccination by approximately 20% to 30%.

Additionally, this study did not include QALYs or DALYs—internationally recognized metrics for health economic evaluation—due to the lack of locally validated utility or disability weights for the elderly population in Guangxi. Instead, we adopted the net benefit and benefit–cost ratio in line with Chinese pharmacoeconomic guidelines. We acknowledge this may limit international comparability and suggest future studies incorporate QALY/DALY metrics as more localized data become available.

## 5. Conclusions

In conclusion, although both the free and self-paid PPV23 vaccination strategies fall short of conventional economic viability thresholds (e.g., *BCR* > 1), the publicly funded strategy demonstrated relatively greater economic efficiency, higher projected health benefits, and a lower *ICER* compared to the self-paid alternative. These findings suggest that, under fiscal constraints, a publicly funded single-dose PPV23 vaccination program may represent a more efficient allocation of health resources than out-of-pocket vaccination. As part of Guangxi’s Healthy Aging Initiative, such a program could serve as a socially equitable and regionally tailored pilot model for vaccine policy innovation in China’s multi-ethnic border regions, contributing to improved population health and better health system performance.

## Figures and Tables

**Table 2:** Cost-effectiveness comparison between free and self-paid PPV23 vaccination strategies.

Strategy	Cost (USD)	Benefit (USD)	Net Benefit (USD)	*BCR*	*ICER*
Free Vaccination	38,231,648	399,654.855	−4,932,513.62	0.075	-
Self-Paid Vaccination	10,954,872	22,260.042	−1,505,616.11	0.015	-
Free vs. Self-Paid	-	-	-	-	10.55
No Vaccination	0	0	0	-	0

## Data Availability

All data sources are indicated in Table 2, and the corresponding author can be contacted.
